# Immune response of macrophages from young and aged mice to the oral pathogenic bacterium *Porphyromonas gingivalis*

**DOI:** 10.1186/1742-4933-7-15

**Published:** 2010-11-29

**Authors:** Yazdani B Shaik-Dasthagirisaheb, Alpdogan Kantarci, Frank C Gibson

**Affiliations:** 1 Department of Medicine, Section of Infectious Diseases, Boston University School of Medicine, Boston, Massachusetts 02118, USA; 2 Department of Periodontology and Oral Biology, Boston University Henry M. Goldman School of Dental Medicine, Boston, Massachusetts 02118, USA

## Abstract

Periodontal disease is a chronic inflammatory gum disease that in severe cases leads to tooth loss. *Porphyromonas gingivalis *(Pg) is a bacterium closely associated with generalized forms of periodontal disease. Clinical onset of generalized periodontal disease commonly presents in individuals over the age of 40. Little is known regarding the effect of aging on inflammation associated with periodontal disease. In the present study we examined the immune response of bone marrow derived macrophages (BMM) from young (2-months) and aged (1-year and 2-years) mice to Pg strain 381. Pg induced robust expression of cytokines; tumor necrosis factor (TNF)-α, interleukin (IL)-6, and IL-10, chemokines; neutrophil chemoattractant protein (KC), macrophage colony stimulating factor (MCP)-1, macrophage inflammatory protein (MIP)-1α and regulated upon activation normal T cell expressed and secreted (RANTES), as well as nitric oxide (NO, measured as nitrite), and prostaglandin E2 (PGE2) from BMM of young mice. BMM from the 2-year age group produced significantly less TNF-α, IL-6 and NO in response to Pg as compared with BMM from 2-months and 1-year of age. We did not observe any difference in the levels of IL-1β, IL-10 and PGE2 produced by BMM in response to Pg. BMM from 2-months and 1-year of age produced similar levels of all chemokines measured with the exception of MCP-1, which was reduced in BMM from 1-year of age. BMM from the 2-year group produced significantly less MCP-1 and MIP-1α compared with 2-months and 1-year age groups. No difference in RANTES production was observed between age groups. Employing a Pg attenuated mutant, deficient in major fimbriae (Pg DPG3), we observed reduced ability of the mutant to stimulate inflammatory mediator expression from BMMs as compared to Pg 381, irrespective of age. Taken together these results support senescence as an important facet of the reduced immunological response observed by BMM of aged host to the periodontal pathogen Pg.

## Background

As humans age, they become more susceptible to a variety of infections including those of the lung, urinary tract, and skin [1]. This increase in infection results in concomitant increase in morbidity and mortality for elderly individuals [1]. The mechanisms underlying age-dependent increase in susceptibility to infection are complex, though decline in immune function and immune senescence is thought to play a central role [2]. Age-related changes in immune fitness have been identified in both the adaptive and innate arms. Effects associated with aging on adaptive immunity include diminished capacity of cells to present antigen, as well as reduced capacity to generate antigen-specific T and B cells [3,4]. Changes in innate immune response include capacity to recognize and respond to pathogens, which may be in part as a result of reduced expression of cell surface receptors, such as the Toll-like receptors (TLRs) [5]. TLRs are a family of receptors that bind conserved microbial structures shared by large groups of pathogens, termed pathogen-associated molecular patterns. Ligation of these receptors with specific pathogen derived antigens initiates intracellular signaling that culminates in coordinated expression of genes that encode molecules such as cytokines and chemokines [6]. Cytokines and chemokines serve diverse roles in many host functions including cell activation, immune function, and communication as well as cell recruitment. Aberrant production of cytokines and chemokines has been observed in aged individuals as compared with young [7].

Periodontal disease is a common chronic oral inflammatory disease [8]. Although complex in pathogenesis, it is commonly thought that erosion of the soft and hard tissue supporting teeth results from bacteria-elicited inflammation. Among the various bacterial species associated with the development of periodontitis, *Porphyromonas gingivalis *(Pg), a Gram-negative anaerobic bacterium suspected to be one of the most important causative agents of the chronic form of this disease [9,10]. Pg produces several virulence factors, including outer membrane vesicles, adhesins, lipopolysaccharide (LPS), hemolysins and proteinases [11-15]. It is established that the innate immune response to this organism and its antigens plays a predominant role in periodontal disease pathogenesis [16]. TLRs have been shown to participate in the recognition of Pg components such as LPS, and fimbriae, which in turn leads to aspects of periodontal disease including cytokine, chemokine production, and oral bone loss [13,15,17]. Epidemiological data from humans has shown Pg in the oral cavity of young adults (20-30 years of age), however clinical onset of periodontal disease is not commonly identified until the 3-4 decade of life. Susceptibility to this disease increases with age [18,19]. Despite evidence supporting early acquisition of Pg and other periodontal pathogens to the oral microflora [18], why adults show onset of periodontal disease later in life is not clearly understood. Employing a macrophage challenge model, in the present study we were interested to determine if the innate immune response to Pg is affected by host age. Our observations identify that BMM from aged mice respond to Pg challenge with reduction in key sets of immune mediators, as compared with BMM obtained from young animals. Data from these studies support that immune senescence may play an important role in the age-dependent pathogenesis of periodontal disease.

## Experimental system

To address the effect of aging on immunological response to periodontal pathogens, we employed an *in vitro *macrophage model and cultured these cells with wild type *Porphyromonas gingivalis *strain 381 (Pg 381), a laboratory strain [20] originally isolated from a patient with chronic periodontitis [21]. Mice were purchased from Jackson Laboratory (Bar Harbor, ME) and aged in accordance with Boston University Institutional Animal Care and Use Committee approvals. Bone marrow derived macrophages (BMM) were generated from young (2-months) and aged (1-year and 2-year) C57BL-6 mice. Bone marrow cells were harvested following sacrifice, and differentiated to BMM for 7 days in RPMI 1640 supplemented with 20% L-929 cell culture conditioned media as source of macrophage colony stimulating factor (M-CSF) [22]. Adhered cells were collected, placed into wells of tissue culture plates at 5 × 10^5 ^cells/mL, and were challenged with anaerobically grown Pg organisms at multiplicity of infection (MOI) of 100 as previously described [20]. BMM were also cultured with a genetically engineered major fimbriae-deficient mutant, Pg DPG3, characterized both *in vitro *and *in vivo *to be virulence attenuated [23-25]. Culture supernatant fluids were collected from unchallenged and Pg-challenged BMM after 24 h of co-culture and the levels of cytokines, chemokines, nitric oxide (measured as nitrite) and PGE2 were determined.

## Results

**Figure 1:**
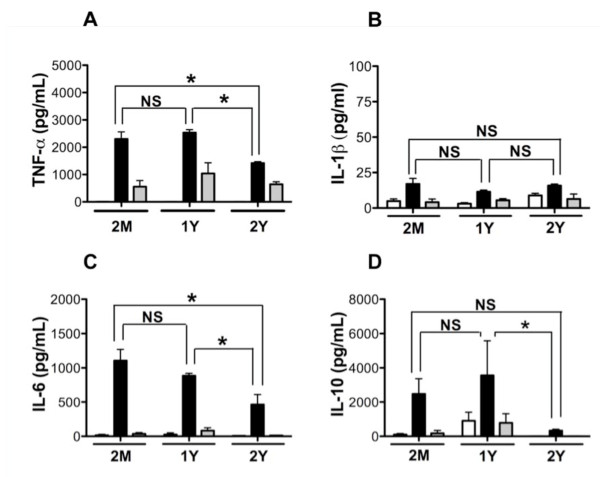
**Age dependent cytokine production from mouse BMM in response to Pg**. Cytokine analysis in cell culture supernatant fluids of mouse BMM from 2-months (n = 6), 1-year (n = 3) and 2-years (n = 3) of age. Cells were cultured with Pg 381 (filled bars), Pg DPG3 (gray bars) at multiplicity of infection (MOI) of 100, or medium only control (open bars) for 24 h. Cytokine levels were determined by multiplex analysis and the data presented as pg/mL mean ± standard error of the mean. **A**. TNF-α; **B**. IL-1β; **C**. IL-6; and **D**. IL-10. Asterisks indicate statistically significant (P < 0.05) as determined by Two way ANOVA with Bonferroni post-test. NS = not significant.

Cytokines are important signaling molecules induced from immune cells in response to microbial infection. Elevated levels of both pro- and anti-inflammatory cytokines have been detected in tissues from patients with periodontitis [26,27]. Experimental studies support that periodontal pathogens such as Pg and its antigens are capable of driving cytokine production from immune cells [28-30]. To determine the levels of cytokines and chemokines in the culture supernatant fluids from BMM-Pg co-cultures, we used the xMAP multiplex immuno assays. Culture supernatant fluids from unchallenged and Pg challenged BMM were added to wells (50 μL/well) of 96 well filter bottom plates and incubated with microsphere beads coated with anti-mouse antibodies against studied targets according to manufacturer's instructions (Invitrogen, Carlsbad, CA). Plates were read with a Luminex 200 multi laser scanner (Luminex, Austin, TX), and data analysis was performed using Bio-Plex Manager software (Bio-Rad, Hercules, CA). The concentration of each analyte was calculated using assay standard curves. We observed that cell culture supernatant fluids from Pg-challenged BMM responded with robust induction of both pro-inflammatory and anti-inflammatory cytokines (Figure 1). Supernatant fluid levels of the pro-inflammatory cytokines TNF-α and IL-6 were similar between BMM of 2-months and 1-year aged mice. BMM from 2-year aged mice exhibited attenuated TNF-α and IL-6 production when compared to the 2-months and 1-year age groups (Figure 1A and 1C). Focusing on another pro-inflammatory cytokine, IL-1β, we observed slightly elevated IL-1β in culture supernatant fluids of BMM cultured with Pg; however, no difference was observed in the IL-1β response to Pg between the age groups (Figure 1B). Next we measured levels of the immunoregulatory/anti-inflammatory cytokine IL-10, a key molecule associated with periodontal disease and host response to Pg [31,32]. We observed that Pg elicited a strong IL-10 response from BMM. Furthermore, the levels of IL-10 were significantly reduced in culture supernatant fluids of BMM from 2-year aged mice as compared to BMM from the 1-year age group (Figure 1D). No statistically significant differences in IL-10 were observed between the 2-months and 2-year age groups, although trend existed (Figure 1D). These cytokine data support that BMM from aged mice exhibited reduced ability to produce both pro-inflammatory and anti-inflammatory cytokines in response to Pg 381 exposure.

**Figure 2:**
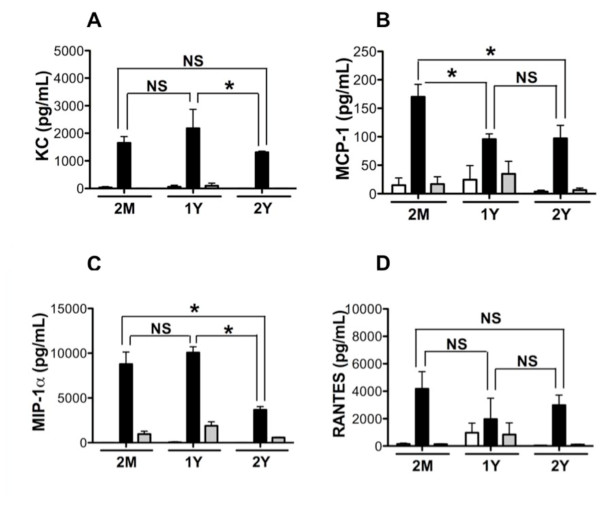
**Chemokine response of young and aged mouse BMM to Pg**. Chemokine levels in cell culture supernatant fluids of mouse BMM from 2-months (n = 6), 1-year (n = 3) and 2-years (n = 3) of age. BMM were challenged with Pg 381 (filled bars), Pg DPG3 (gray bars) at multiplicity of infection (MOI) of 100, or medium only control (open bars) for 24 h. Chemokine levels were determined by multiplex analysis and the data presented as pg/mL mean ± standard error of the mean. **A**. KC; **B**. MCP-1; **C**. MIP-1α; and **D**. RANTES. Asterisks indicate statistically significant (P < 0.05) as determined by Two way ANOVA with Bonferroni post-test analysis. NS = not significant.

Chemokines are a large family of host secreted glycoproteins that possess roles in a myriad of host functions including recruitment of specific sets of immune cells to site of infection [33]. In periodontal disease, chemokines have been detected in tissues of patients, and support chemotaxis and activation of inflammatory cells that comprise the periodontal lesion [34,35]. Based on the clear association between cellular infiltrate and periodontal disease, we measured the levels of chemokines; KC, MCP-1, MIP-1α, and RANTES produced by BMM in response to Pg challenge to determine if aging affects the ability of macrophages to produce these chemokines. BMM responded to Pg challenge with robust expression of all chemokines measured regardless of age. We observed that BMM from young mice expressed high levels of MCP-1 in response to Pg, while levels from the 1-year group were significantly reduced (Figure 2B). No other significant differences were observed in chemokine expression between 2-month and 1-year of age (Figure 2). BMM from 2-year group showed significant reduction in the levels of MIP-1α in response to Pg challenge compared with 2-months age group (Figure 2C), whereas levels of KC significantly reduced in BMM from 2-year group compared with 1-year age group (Figure 2A). Although trends appeared to exist, RANTES expression following Pg 381 challenge was similar between age groups (Figure 2D). This observed profile of chemokine expression complements our cytokine expression data indicating that BMM from aged hosts display attenuation in the levels of innate immune mediators to Pg 381 and the magnitude of this reduced ability to produce chemokines increases with age.

**Figure 3:**
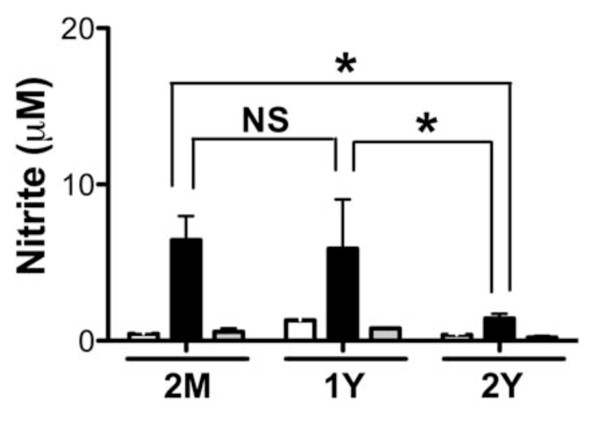
**Nitric oxide levels from young and aged mouse BMM in response to Pg**. NO present in cell culture supernatant fluids from mouse BMM of 2-months (n = 6), 1-year (n = 3) and 2-years (n = 3) of age. BMM were cultured with Pg 381 (filled bars), Pg DPG3 (gray bars) at multiplicity of infection (MOI) of 100, or medium only control (open bars) for 24 h. Secreted NO levels were inferred by measuring μM nitrite levels using Greiss reaction as previously described [36]. Asterisks indicate statistically significant (P < 0.05) as determined by Two way ANOVA with Bonferroni post-test analysis. NS = not significant.

Next we examined the levels of nitric oxide (NO) in culture supernatant fluids from BMM challenged *in vitro *with Pg, measured as total nitrite by Griess reaction [36]. Inflammatory cells such as macrophages produce NO in response to bacterial challenge. The enzyme inducible nitric oxide synthase (iNOS) is responsible for NO production. Loss in the ability to fully express NO by alveolar macrophages in response to *Listeria monocytogenies *has been linked to aging, and likely contributes to host response to infection [37]. NO possesses bactericidal activity against a wide range of bacteria including Pg [38,39]. Mice deficient in the enzyme iNOS show impaired ability to clear Pg [40,41]. Also mice deficient in iNOS production showed increased tissue damage with Pg challenge [42]. Thus, we were interested to determine if age is associated with macrophage ability to produce NO in response to Pg. Measuring the stable product nitrite as a proxy for NO expression, we observed that BMM cultured with Pg produced high levels of NO as compared with unchallenged control cells. NO levels in culture supernatant fluids were similar between 2-month and 1-year BMM; however, BMM from 2-year age group presented with significantly reduced NO levels in comparison to 2-months and 1-year age groups (Figure 3). Our results suggest that BMM from aged mice (2-years of age) exhibit attenuated capacity to produce NO as compared with BMM from young mice in response to the periodontal pathogen Pg 381.

**Figure 4:**
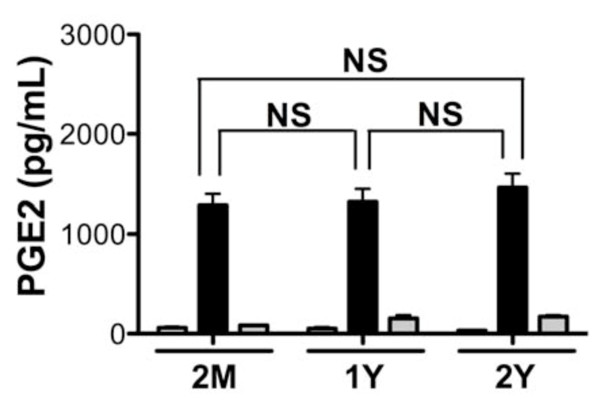
**Prostaglandin E2 levels from young and aged mouse BMM in response to Pg**. PGE2 present in cell culture supernatant fluids of mouse BMM from 2-months (n = 6), 1-year (n = 3) and 2-years (n = 3) of age. Cells were challenged with Pg 381 (filled bars), Pg DPG3 (gray bars) at multiplicity of infection (MOI) of 100, or medium only control (open bars) for 24 h. PGE2 levels were measured by ELISA and the data presented as pg/mL mean ± standard error of the mean. Statistically was determined by Two way ANOVA with Bonferroni post-test analysis. NS = not significant.

PGE2 is an immunomodulatory product of the arachidonic acid pathway that is secreted by immune cells in response to microbial challenge. Prostaglandins including PGE2 possess both pro- and anti-inflammatory activities, and can affect bone metabolism [43]. Elevated PGE2 levels have been detected in periodontal disease and are thought to play a key role in the progression of this disease [44]. To determine if the magnitude of prostaglandin production by BMM in response to Pg challenge is influenced by aging, we measured PGE2 levels in the culture supernatant fluids of BMM cultured with Pg by ELISA (R&D Systems, Minneapolis, MN). Pg induced robust expression of PGE2 in BMM from all age groups in comparison to unchallenged BMM. Levels of Pg-elicited PGE2 were similar among the age groups (Figure 4). These results indicate that Pg elicits robust expression of PGE2 from BMM independent of age.

As epidemiological data support that incidence of periodontal disease increases with age [18,19], and our data using wild type Pg indicated that BMM from aged mice displayed an overall trend of reduced ability to produce immune mediators as compared to that of young mice. We speculated that one possibility for the increased incidence of periodontal disease in aged populations may reflect the reduced function of immune response and an attenuated strain of Pg can induce immune response similar to a virulent Pg strain. To test this, we challenged BMM with wild type Pg 381 and a genetically attenuated mutant Pg strain that lacks major fimbriae, Pg DPG3 [45]. Major fimbriae are involved in Pg binding to cells [46,47]. This mutation has identified roles for Pg major fimbriae in stimulation of cytokines and chemokines from cells [23,48], and oral bone loss [49]. This attenuated organism did not stimulate expression of any of the cytokines measured from young or aged BMM as robustly as wild type strain, Pg 381 (Figure 1A, B, C and 1D). Unexpectedly, we observed that despite the mutation, Pg DPG3 did induce a mild TNF-α response as compared to unchallenged control that occurred independent of age (Figure 1A). Focusing on chemokine expression, we observed that Pg DPG3 stimulated BMM also to produce MIP-1α as compared with unchallenged control, like TNF-α, these levels were significantly less than that observed with wild type Pg challenge (Figure 2C). KC, MCP-1, and RANTES production by BMM in response to Pg DPG3 challenge was not evident and levels of these mediators resembled the unchallenged controls (Figure 2A, B and 2D). Similarly Pg DPG3 failed to elicit NO and PGE2 production by BMM (Figure 3 and 4). These results collectively indicate that BMM exhibit a deprived innate immune response to an attenuated Pg mutant, suggesting, at least under these conditions, that aging does not contribute to BMM mounting vigorous immune responses to attenuated Pg similar to the wild type Pg 381.

## Discussion

Periodontal disease is increasingly more common in adults as they age, suggesting that aging influences periodontal disease [18,19,50,51]. Despite this clear epidemiological connection, the mechanisms underlying aging and increased incidence/progression of periodontal disease are poorly understood; however, inflammatory response contributes significantly to periodontitis-associated tissue destruction. In the present study we examined the innate immune responses of BMM from 2-months (young), 1-year and 2-years (aged) mice in response to Pg 381 and an attenuated mutant Pg DPG3 *in vitro*. By measuring levels of cytokines, chemokines and other innate immune mediators in culture supernatant fluids from these cells in response to Pg challenge, despite some mediators, it is clear that BMM from aged mice display a reduced response to Pg 381 (Figure 1, 2, 3 and 4). It is evident from our data that BMM respond less vigorously to the attenuated mutant Pg DPG3 compared to Pg 381, and this occurred independent of age. This data indicate that major fimbriae is involved in Pg-elicited innate immune response of murine BMM (Figure 1, 2, 3 and 4) and that attenuated Pg strain responded similar manner to the BMM independent of age. Together these data support that aging contributes to attenuated expression of cytokine, chemokine and other innate immune response mediators by murine BMM to Pg challenge *in vitro*. From these results, focusing on expression profiles of a limited, but highly relevant set of markers, as well as using only one cell type (BMM), it suggest that immunological senescence may participate in limiting host response to periodontal pathogen Pg and thus compromise immune function with age. These data are in agreement with the clinical observation that susceptibility to periodontal disease increases with age [52,53].

Recently it has been shown that macrophages from aged mice exhibit reduced production of cytokines to bacterial antigens including LPS and polysaccharide as compared to macrophages from young mice [54,55]. It is not completely understood the reason behind age dependent reduction in macrophage function; however, TLR expression is reduced on macrophages from old mice as compared with young, and thus may be responsible for the reduced cytokine levels expressed by macrophages from aged mice [5,56]. Although we did not define TLR expression on our macrophage population, it is possible that a TLR-dependent mechanism may be involved in host response to Pg. Indeed, both TLR2 and TLR4 have been implicated in recognition of live Pg [57] and several of its antigens including LPS and major fimbriae [17,58] albeit in cells from young mice. Another potential mechanism that could be at bacterial advantage in aged populations is increased survival of pathogenic microorganisms in aged mice in comparison with young group [59,60]. A recent study using peritoneal macrophages from BALB/c mice showed no differences in the levels of either IL-1β or TNF-α proteins between macrophages of young (8-10 weeks) and aged (≥ 18 months) groups in response to Pg; moreover, measured levels of IL-6 were significantly reduced in the aged group compared with young group [61]. However there was no significant difference in the transcript levels of these genes between macrophages from young and aged mice. Similar levels of secreted nitrite were also observed between peritoneal macrophages of young and old mice, cultured with Pg [61]. Several of our findings in the current study differ from those observed previously for host response of young and aged macrophages to Pg. In the present study, we measured significantly reduced levels of TNF-α, and nitrite secreted from BMM of the aged (2-years) group as compared to the young group (2-months); whereas, Liang *et al*. [61] observed no significant difference in the levels of these molecules. Molecules where expression profiles were in agreement between our current study and this previous study [61] includes IL-6, which was reduced in aged macrophages, and IL-1β were no difference in expression between young and aged was observed. Although we do not understand why our data differ based on statistical interpretations from that of the previous study, clearly differences in experimental design between these two studies might have contributed. We employed C57BL/6 mice, whereas Liang *et al*. [61] used BALB/c mice. In addition, we employed Pg strain 381 while the previous study focused on Pg strain 33277.

Indeed, the choice of both mouse and Pg strains used may play importantly in the magnitude of the final measurements obtained, as it has been reported that these two variables can contribute substantially to data out-comes [62-64]. Regardless of the statistical interpretation, our results support the overall assessment of the study by Liang *et al*. [61] that aging leads to reduction in macrophage function in response to Pg. Observations from present study identify that BMM from aged mice respond to Pg challenge with reduction in key sets of immune mediators, as compared with BMM obtained from young mice.

In conclusion, our data from BMM of young and aged mice provides evidence of attenuated innate immune mediator expression to Pg challenge and suggest that immunological senescence is an important feature of the aging process that influences host response to periodontal pathogens. Further studies are clearly necessary to identify and better characterize the mechanisms underlying reduced immunological response of aged population to bacteria such as Pg in the context of periodontal disease pathogenesis.

## Abbreviations

BMM: mouse bone marrow derived macrophages; IL: interleukin; KC: neutrophil chemoattractant or CXCL1; M-CSF: macrophage colony stimulating factor; MCP-1: monocyte chemotactic protein-1; MIP-1α: macrophage inflammatory protein-1alpha; MOI: multiplicity of infection; NO: nitric oxide; Pg: *Porphyromonas gingivalis*; PGE2: prostaglandin E2; RANTES: regulated upon activation normal T cell expressed and secreted; TLRs: toll like receptors; TNF-α: tumor necrosis factor alpha.

## Competing interests

The authors declare that they have no competing interests.

## Authors' contributions

YBS and FCG designed experiments, YBS performed experimentation, data analysis and preparation of manuscript were performed by YBS, AK, and FCG. All authors have read and approved the final manuscript.
